# Focal segmental glomerulosclerosis, excluding atypical lesion, is a predictor of renal outcome in patients with membranous nephropathy: a retrospective analysis of 716 cases

**DOI:** 10.1186/s12882-019-1498-4

**Published:** 2019-08-22

**Authors:** Hong-Guang He, Chao-Qing Wu, Kun Ye, Chun Zeng, Yi-Yun Huang, Shu-Wen Luo, Wu Yin, Qiu-Rong Ye, Xiao-Mei Peng

**Affiliations:** 1 Department of Nephrology, The People’s Hospital of Guangxi Zhuang Autonomous Region, 6 Taoyuan Road, Qingxiu District, Nanning, 530000 China; 2 Department of Pathology, The People’s Hospital of Guangxi Zhuang Autonomous Region, 6 Taoyuan Road, Qingxiu District, Nanning, 530000 China

**Keywords:** Idiopathic membranous nephropathy, Focal segmental glomerulosclerosis, Prognostic factors, Pathology, Atypical focal segmental lesion

## Abstract

**Background:**

Focal segmental lesions (FSLs) are not uncommon in idiopathic membranous nephropathy (IMN). The reported percentage of IMN patients with focal segmental glomerulosclerosis (FSGS) lesions varies widely between studies. The objective of this study was to differentiate atypical FSL (aFSL) from typical FSGS in IMN and to analyse the clinicopathological predictors of primary outcome of IMN patients.

**Methods:**

A total of 716 patients with biopsy-proven IMN between January 1, 2007 and December 31, 2017 were enrolled in the study. An atypical focal segmental lesion was defined as pure synechia, segmental hyperplasia of podocytes or thickening of the GBM accompanied by proliferation of the mesangial matrix, and absence of typical FSGS. The patients were divided into three groups: patients without FSL (FSL^−^), patients with typical FSGS (FSGS^+^), and patients with aFSL (aFSL^+^).The primary outcome was a 50% decline in the initial estimated glomerular filtration rate or end-stage renal disease (ESRD) incidence. Secondary outcomes included all-cause death and ESRD.

**Results:**

FSGS was present in 174 patients, while aFSL was noted in 161 patients. Systolic blood pressure was higher in both aFSL^+^ group and FSGS^+^ groups compared with the FSL^−^ group. IMN patients without FSL and with aFSL had lower serum creatinine levels than IMN patients with FSGS. Both the FSGS^+^ and aFSL^+^ groups had higher levels of proteinuria and lower albumin levels than the FSL^−^ group. Renal tissue lesions, including tubulointerstitial fibrosis, glomerular obsolescence, and vascular sclerosis were significantly more severe in the FSGS^+^ group. Cox multivariate analysis showed that older age ≥ 60 years, eGFR< 60 ml/(min·1.73m^2^), tubulointerstitial fibrosis area ≥ 15% and FSGS at biopsy were independent risk factors for the primary outcome.

**Conclusions:**

No significant difference in outcome was found between the FSL^−^ and aFSL^+^ groups, although the patients with aFSL had lower levels of serum albumin and eGFR, higher level of urinary protein, more severe renal lesions with proliferation of the mesangial area,tubulointerstitial fibrosis and vascular sclerosis. FSGS, excluding atypical lesions, was an independent predictor of the primary outcome.

## Background

Idiopathic membranous nephropathy (IMN) is one of the most common causes of adult-onset nephrotic syndrome both in Caucasian and the Chinese individuals [1, 2], and China has witnessed an increasing prevalence of IMN among patients with primary glomerulonephritis, from 7% in 1997–1999 to 23% in 2009–2011 [2]. In 1977, Ehrenreich and Churg [3] observed lesions of focal sclerosis in cases of membranous nephropathy (MN),and some studies have indicated that focal segmental glomerulosclerosis (FSGS) is a risk factor for poor prognosis or an independent indicator of poor prognosis [4–8]. An international group of renal pathologists convened at Columbia University to reach a consensus regarding the pathologic classification of FSGS [9]; however, whether FSGS is an independent predictor in IMN remains unresolved [10–13]. The percentage of IMN patients with FSGS lesions differs in the literature, ranging from 2.5 to 41.7% [4, 7, 12–15],and in addition to geographical distribution, enrolment methods and ethnicity, the different definitions of FSGS lesions in IMN used in clinical practice may account for this variability.

In previous studies, segmental lesions in IMN included perihilar, not-otherwise specified (NOS), and tip variant [7], while the atypical segmental lesion (aFSL) described in the present study, which includes synechia [4], segmental hyperplasia of podocytes, and segmental glomerular basement membrane (GBM) thickening and/or with proliferation of mesangial matrix of the segmental tuft is also commonly seen in IMN.

In this study, we aimed to differentiate atypical focal segmental lesion (aFSL) from typical FSGS in IMN, ascertain the clinicopathological characteristics of IMN with aFSL, and analyse the clinicopathological characteristics and outcomes of IMN patients with aFSL and FSGS.

## Methods

### Patient selection

We included patients with a diagnosis of IMN, who are biopsied between January 1, 2007 and December 31, 2017 in our hospital, excluding patients with diagnoses such as lupus nephritis, MN related to hepatitis B virus, malignancy, metal poisoning, or other diseases associated with secondary MN. The enrolled patients were treated with angiotensin-converting enzyme (ACE) inhibitors or angiotensin receptor blockers, corticosteroids, cyclophosphamide, cyclosporine, tacrolimus, azathioprine, *Tripterygium wilfordii* [16], and mycophenolate mofetil. All patients were ≥ 14 years of age at the time of renal biopsy. All renal biopsies were evaluated by light and immunofluorescence microscopy, ultrastructural evaluation was performed if necessary. Follow-up started at the time of biopsy and either continued until December 2018 or ended at the time of death or development of end-stage renal disease (ESRD).

### Clinical parameters

For the enrolled patients, age, sex, duration of disease before biopsy, levels of serum creatinine and albumin, 24-h proteinuria content, systolic and diastolic blood pressures were recorded at the time of biopsy.

The Chronic Kidney Disease Epidemiology Collaboration (CKD-EPI) equation was used to estimate glomerular filtration rate (eGFR) [17]. The CKD was classified based on the KDIGO 2012 Clinical Practice Guideline [18]. Hypertension was defined as blood pressure exceeding 140 over 90 mmHg or currently receiving antihypertensive therapy.

### Histopathologic parameters

**Fig. 1:**
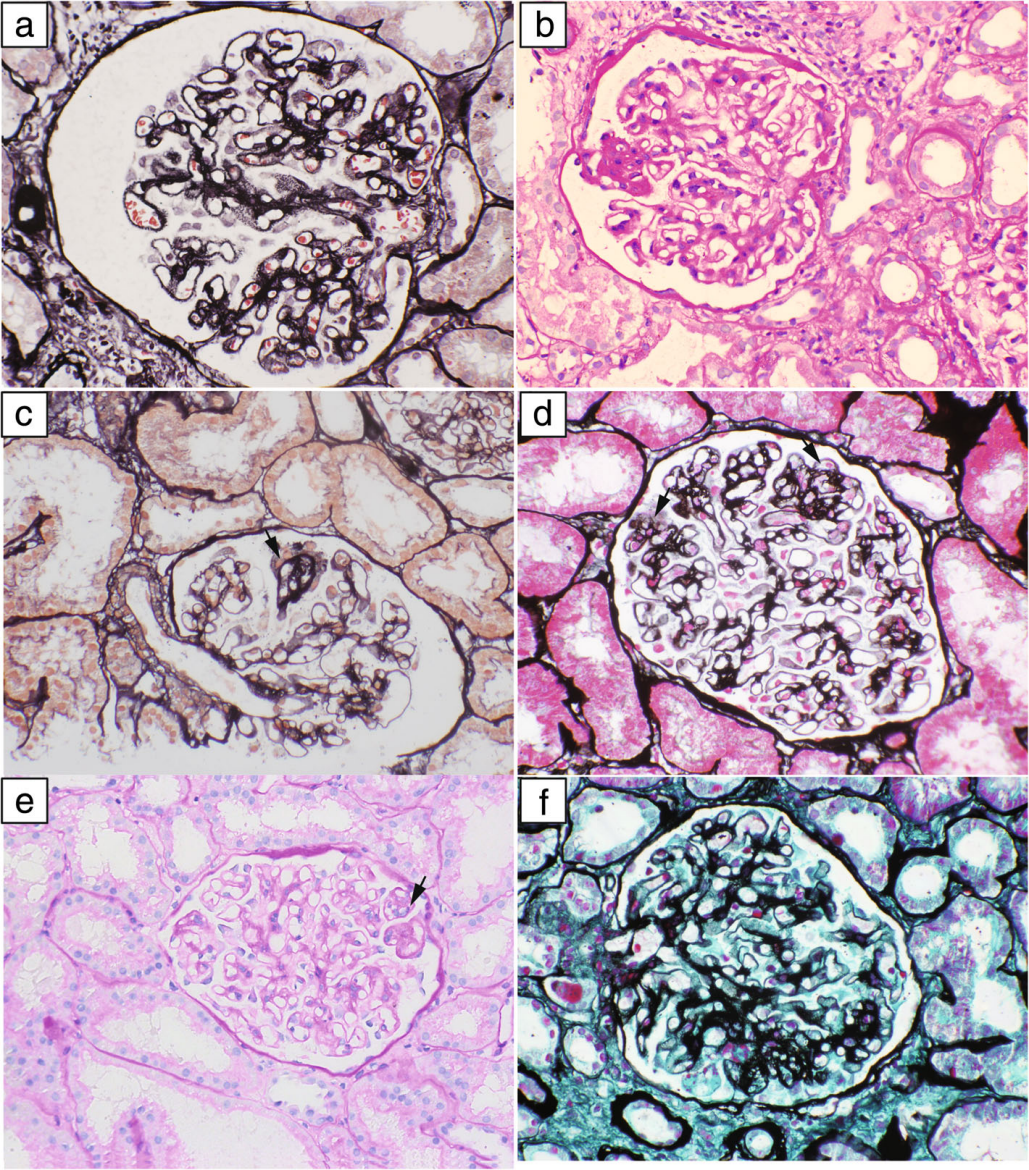
Early focal segmental lesions in IMN patients: **a** Pure synechia to Bowman’s capsule,with glomerular enlargement;(PASM;original magnification× 400.) **b** Synechia at the tip area,accompanied with proliferation of mesangial matrix; (PAS;original magnification× 400.) **c** Segmental thickening of GBM, accompanied with hyperplasia of podocytes (arrow); (PASM;original magnification× 400.) **d** Proliferation of extracellular matrix of segmental tuft, with slight hyperplasia of podocytes and proliferation of endothelial cells (arrows);(PASM;original magnification× 400.) **e** Segmental thickening of GBM,accompanied with segmental proliferation of endothelial cell (arrow);(PAS;original magnification× 400.) **f** Segmental proliferation of extracellular matrix of segmental tuft, with hyperplasia of podocytes and proliferation of endothelial cells.(PASM;original magnification× 400)

Renal biopsy specimens including at least eight glomeruli were analysed in this study. Renal tissue specimens were examined by two pathologists with no knowledge of the patients’ clinical condition to establish the diagnosis by standard pathologic methods alone. The pathological features under light microscopy, stage, global sclerosis, segmental sclerosis, tubulointerstitial fibrosis, and arteriosclerosis were collected. FSL was graded as present or absent, including FSGS and aFSL. AFSL was defined as follows: 1) pure synechia (Fig. 1a), and synechia at the tip pole, and/or accompanied by proliferation of matrix (Fig 1b) also was also classified in aFSL in present study; 2) Hyperplasia of podocytes (Fig. 1c, arrow), which may be accompanied by segmental thickening of the GBM or proliferation of mesangial matrix; 3) Proliferation of the extracellular matrix of the segmental tuft (Fig. 1d, f), or thickening of the GBM (Fig. 1e); often accompanied by segmental endothelial cell hyperplasia (Fig. 1 e, f); and 4) absence of typical FSGS, with no accumulation of inframembranous hyaline, no collapsing tuft, and without foam cells occluding the lumina. FSGS lesions were categorized according to the Columbia FSGS classification system [9], but synechia at the tip pole (and/or accompanied with proliferation of matrix), was classified as aFSL in the present study, in order to coincide with the definition of other locations..

Based on this classification, the subjects were divided into three groups: patients without focal segmental lesion (FSL^−^), with FSGS (FSGS^+^), and with aFSL,but not co-occurring with FSGS (aFSL^+^). Tubulointerstitial fibrosis was defined as increased extracellular matrix separating tubules in the cortical area and was recorded according to the area of fibrosis. The score for degree of vascular sclerosis was based on the most severe lesion seen in either arterioles or arteries [11].

### Study outcomes

The primary end point was the composite of ESRD (defined by persistent eGFR< 15 ml/min per 1.73m^2^, start of chronic dialysis, or preemptive kidney transplantation) or a 50% reduction in eGFR over follow-up. Secondary outcomes included all-cause death, and ESRD.

### Statistical analysis

Normally distributed variables were expressed as the mean ± standard deviation and compared using one-way analysis of variance. Nonparametric continuous variables were expressed as medians (interquartile ranges) and compared using the Kruskal–Wallis test. Categorical variables were expressed as percentages and compared using the χ^2^-test or Fisher exact test. The cumulative probabilities of event-free survival for outcomes were determined using the Kaplan-Meier method and log-rank tests. The independent risk factors for outcomes were analysed with the Cox proportional hazards model. All *P-*values were two-tailed and values< 0.05 were considered to indicate statistical significance. The confidence interval included 95% of predicted values. Statistical analyses were performed using SPSS statistical software (version 23.0, SPSS, Chicago, IL).

## Results

### Baseline characteristics

A total of 716 patients with IMN were included in this study. The mean age at diagnosis was 49 ± 14 years and the median follow-up was 20(interquartile range:8–35) months. The median serum creatinine level was 77(interquartile range:62–91) μmol/L. There were 68 patients with an eGFR of < 60 mL/min per 1.73 m^2^ and 388 with an eGFR of > 90 mL/min per 1.73 m^2^.The median proteinuria value was 4.8 g/day (interquartile range: 2.7–7.5 g/day),and 150 patients had proteinuria of > 8.0 g/day.

A comparison of demographic and baseline clinicopathological data among the FSGS^+^, aFSL^+^, and FSL^−^ IMN patients is summarized in Table 1. FSGS was present in 174 patients, while aFSL was noted in 161 patients. Compared with FSL^−^ and aFSL^+^ patients, patients with combined FSGS lesions had higher systolic blood pressure, diastolic blood pressure, and serum creatinine level and a lower GFR. Compared with the FSL^−^ group, both the FSGS^+^ and aFSL^+^ groups had significantly lower albumin, and higher 24-h urine protein.

**Table 1:** Comparison of the clinicopathological parameters of patients among the three groups at baseline

Parameters	FSL^−^	aFSL^+^	FSGS^+^
n	381 (53.2%)	161 (22.5%)	174 (24.3%)
Male, n (%)	193 (50.7%)	92 (57.1%)	109 (62.6%)^†^
Age (years)	49 ± 14	48 ± 15	52 ± 14^†§^
Age ≥ 60,*n*(%)	91 (23.9%)	40 (24.8%)	57 (32.8%)
SBP (mm Hg)	130 (119,147)	136 (123,154)^*^	147 (132,162)^†§^
DBP (mm Hg)	80 (72,88)	80 (74,91)	86 (77,95)^†§^
Hypertension, *n* (%)	158 (41.5%)	86 (53.4%)	118 (67.8%)^†§^
Duration of disease before biopsy (months)	2 (1,5)	2 (1,7)	4 (1,9) ^†^
Albumin(g/L)	24.4 (20.7,28.9)	21.7 (18.5,26.9)^*^	20.9 (17.0,24.4)^†^
Urine protein content(g/24 h)	4.0 (2.3,6.6)	5.4 (3.4,8.1)^*^	5.6 (3.6,8.8)^†^
Serum creatinine (μmol/L)	72 (59,83)	78 (64,92)^*^	85 (69,103)^†§^
eGFR (ml/min per 1.73m^2^)	98 (83,110)	91 (77,109)^*^	81 (64,102)^†§^
CKD ≥ 3stage	13 (3.4%)	18 (11.2%)	37 (21.3%)^†§^
Immunosuppressants	289 (75.9%)	136 (84.4%)	151 (86.8%)^†^
Stage		*	†
I	131 (34.4%)	32 (19.9%)	26 (14.9%)
II	214 (56.2%)	88 (54.7%)	104 (59.8%)
III	35 (9.2%)	40 (24.8%)	42 (24.1%)
IV	1 (0.3%)	1 (0.6%)	2 (1.1%)
Global glomerulosclerosis (%)	0 (0,6.3)	2.3 (0,8.4)	5.8 (0,12.6)^†§^
Proliferation of mesangial area		*	†§
0	191 (50.1%)	58 (36%)	40 (23%)
1	171 (44.9%)	85 (52.8%)	106 (60.9%)
2	19 (5%)	17 (10.6%)	25 (14.4%)
3	0 (0%)	1 (0.6%)	3 (1.7%)
Tubulointerstitial fibrosis(%)	2 (0,5)	3 (2,8)^*^	8 (3,18.5)^†§^
Vascular sclerosis			†§
0	105 (27.6%)	41 (25.5%)	22 (12.6%)
1	211 (55.4%)	88 (54.7%)	97 (55.7%)
2	56 (14.7%)	28 (17.4%)	42 (24.1%)
3	9 (2.4%)	4 (2.5%)	13 (7.5%)

Abbreviations: *SBP* Systolic blood pressure, *DBP* Diastolic blood pressure, *eGFR* Estimated glomerular filtration rate; Datas are presented as n (%), mean ± s.d. or median (interquartile range)

^*^ *P* < 0.05 between the FSL^−^ and aFSL^+^ groups;^†^ *P* < 0.05 between the FSL^−^ and FSGS^+^ groups;^§^ *P* < 0.05 between the aFSL^+^ and FSGS^+^ group

Of the 174 patients with FSGS lesions, 70 presented with tip lesions, 73 with NOS lesions, 23 with perihilar lesions, 3 with cellular lesions, and 5 with collapsing lesions; Of the 161 patients with atypical focal segmental lesions, 95 presented with synechia lesions,19 with segmental hyperplasia of podocytes (without synechia lesions), and 47 with segmental thickening of GBM, accompanied by proliferation of the mesangial matrix (without hyperplasia of podocytes or synechia lesions).

The percentage of obsolescent glomeruli was highest in the group of patients with FSGS (vs. FSL^−^ group, *P* < 0.001; vs. aFSL^+^ group, *P =* 0.001). A significant increase in the severity of vascular sclerosis lesions was found in biopsy specimens in group FSGS^+^ (vs. aFSL^+^, *P* = 0.002; vs. FSL^−^, *P <* 0.001). Furthermore, a significant increase in severe tubulointerstitial fibrosis was also found in biopsy specimens in group FSGS^+^(vs. aFSL^+^, *P <* 0.001;vs. FSL^−^, *P <* 0.001) and aFSL^+^(vs. FSL^−^, *P <* 0.001). Regarding the staging of membranous lesions, a later stage was observed in the FSGS^+^ group (*P <* 0.001) and in the aFSL^+^ group (*P <* 0.01) compared with the FSL- group (Table 1).

### Outcomes

Two hundred eighty-nine (75.9%) patients in the FSL^−^ group, 136 (84.4%) in the aFSL^+^ group, and 151 (86.8%) in the FSGS^+^ group were treated with immunosuppressive therapy (Table 1). At the end of follow-up, 44 patients (6.1%) experienced a 50% decline in eGFR (including progression to ESRD), of which 27(3.8%) progressed to ESRD. Twenty-four patients (3.4%) died; causes of death included severe infection (12/25), pulmonary embolism (1/19), and cardio-cerebral vascular events (11/25).

The significance of each factor affecting the primary outcome is shown in Table 2. Among the clinical parameters, old age (≥60 years old), hypertension, increased serum creatinine (≥134umol/L), and eGFR (< 60 ml/min per 1.73 m^2^) were significant risk factors for primary outcome by univariate analysis. However, male sex, low level of albumin, and high level of 24-h urine protein were not identified as risk factors in our analysis. By univariate analysis, a high incidence of global glomerulosclerosis (present in > 5% of total glomeruli), and focal segmental lesions (including both atypical focal segmental lesions and typical FSGS lesions), the presence of FSGS lesions alone, tubulointerstitial fibrosis occupying ≥15% of the total specimen area, proliferation of mesangial area, and vascular sclerosis were significant risk factors for primary outcome (Table 2).

**Table 2:** Univariate analysis of risk factors for progression to primary outcome– Cox proportional hazards model

Parameters	*P* value	HR	HR(95% CI)
Male	0.199	1.505	0.806–2.807
Age ≥ 60 years	< 0.001	4.430	2.406–8.157
Hypertension	< 0.001	4.591	2.134–9.878
Albumin(g/L)			
≥ 30			1.00 (referent)
20–29.9	0.965	1.020	0.414–2.517
≤ 19.9	0.289	1.674	0.646–4.339
Urine protein content(g/24 h)			
< 3.9			1.00 (referent)
4–7.9	0.962	1.017	0.511–2.023
≥ 8	0.258	1.549	0.725–3.311
Serum creatinine≥134(umol/L)	< 0.001	16.888	8.164–34.932
eGFR< 60(ml/min per 1.73 m^2^)	< 0.001	9.509	5.178–17.460
FSL (FSGS,aFSL = 1,FSL^−^ = 0)	< 0.001	3.822	1.961–7.447
FSL			
FSL^−^			1.00 (referent)
FSGS	< 0.001	6.902	3.493–13.635
aFSL	0.914	0.939	0.302–2.919
Stage			
I			1.00 (referent)
II	0.31	1.455	0.706–2.998
III and IV	0.237	1.705	0.704–4.133
Global glomerulosclerosis (> 5%)	< 0.001	5.212	2.680–10.136
Proliferation of mesangial area			
0			1.00 (referent)
1	0.034	2.095	1.059–4.147
2 and 3	0.061	2.741	0.956–7.858
Tubulointerstitial fibrosis (area ≥ 15%)	< 0.001	7.368	4.050–13.404
Vascular sclerosis			
0			1.00 (referent)
1	0.036	4.725	1.104–20.223
2	0.002	10.617	2.440–46.203
3	< 0.001	30.771	6.141–154.175

Abbreviations: *HR* Hazard ratio, *CI* Confidence interval; Datas are presented as *n* (%), or median (interquartile range)

By multivariate analysis, in model 1, old age(≥60 years old), eGFR< 60 ml/min per 1.73 m^2^, tubulointerstitial fibrosis area ≥ 15% were significant risk factors for progression to primary outcome, while focal segmental lesions (including both atypical focal segmental lesions and typical FSGS lesions) was not independent risk factor. However, in model 2, old age(≥60 years old) (HR, 2.870; 95% CI, 1.519–5.424; *P =* 0.001), eGFR< 60 ml/min per 1.73 m^2^ (HR, 2.925; 95% CI, 1.441–5.936; *P =* 0.003;), FSGS lesions (HR, 2.471; 95% CI, 1.113–5.486; *P =* 0.026), tubulointerstitial fibrosis area ≥ 15%(HR, 2.553; 95% CI, 1.248–5.224; *P =* 0.010) were significant independent risk factors for progression to primary outcome (Table 3). Twenty-eight patients (16.1%) in the FSGS^+^ group reached the primary outcome, compared with 12 patients (3.1%) in the FSL^−^ group(*P <* 0.001) and 4 patients (2.5%)in the aFSL^+^ group(*P <* 0.01). Sixteen patients (9.2%) in the FSGS^+^ group reached the ESRD outcome compared with 9 patients (2.4%) in the FSL^−^ group(*P <* 0.001) and 2 patients (1.2%) in the aFSL^+^ group (*P <* 0.01). Nine patients (2.4%) in the FSL^−^ group reached the all-cause death outcome compared with 9 patients (5.2%) in the FSGS^+^ group and 6 patients (3.7%) in the aFSL^+^ group.

**Table 3:** Mutivariate analysis of risk factors for progression to primary outcome– Cox proportional hazards model

Parameters	*P* value	HR	HR(95% CI)
Model 1:FSGS^+^ and aFSL^+^ groups as one group (FSL^+^ group)			
Age ≥ 60 years	0.003	2.602	1.373–4.930
Hypertension	0.127	1.874	0.837–4.192
eGFR< 60(ml/min per 1.73m^2^)	0.009	2.632	1.279–5.415
FSL (FSGS,aFSL = 1,FSL^−^ = 0)	0.251	1.557	0.731–3.320
Global Glomerulosclerosis (> 5%)	0.278	1.548	0.703–3.410
Proliferation of mesangial (stage1,2,3 = 1)	0.648	1.178	0.582–2.383
Tubulointerstitial fibrosis (area ≥ 15% = 1)	0.003	3.008	1.470–6.156
Vascular sclerosis (stage1,2,3 = 1)	0.267	2.293	0.530–9.921
Moldel 2:FSGS^+^ and aFSL^+^ groups as two individual groups			
Age ≥ 60 years	0.001	2.870	1.519–5.424
Hypertension	0.182	1.741	0.771–3.930
eGFR< 60(ml/min per 1.73m^2^)	0.003	2.925	1.441–5.936
FSL			
FSL^−^			1.00 (referent)
FSGS	0.026	2.471	1.113–5.486
aFSL	0.260	0.503	0.152–1.663
Global Glomerulosclerosis (> 5%)	0.338	1.467	0.669–3.215
Proliferation of mesangial (stage 1,2,3 = 1)	0.894	1.049	0.518–2.122
Tubulointerstitial fibrosis (area ≥ 15% = 1)	0.010	2.553	1.248–5.224
Vascular sclerosis (stage 1,2,3 = 1)	0.351	2.014	0.462–8.770

Abbreviations: *HR* Hazard ratio, *CI* Confidence interval

**Fig. 2:**
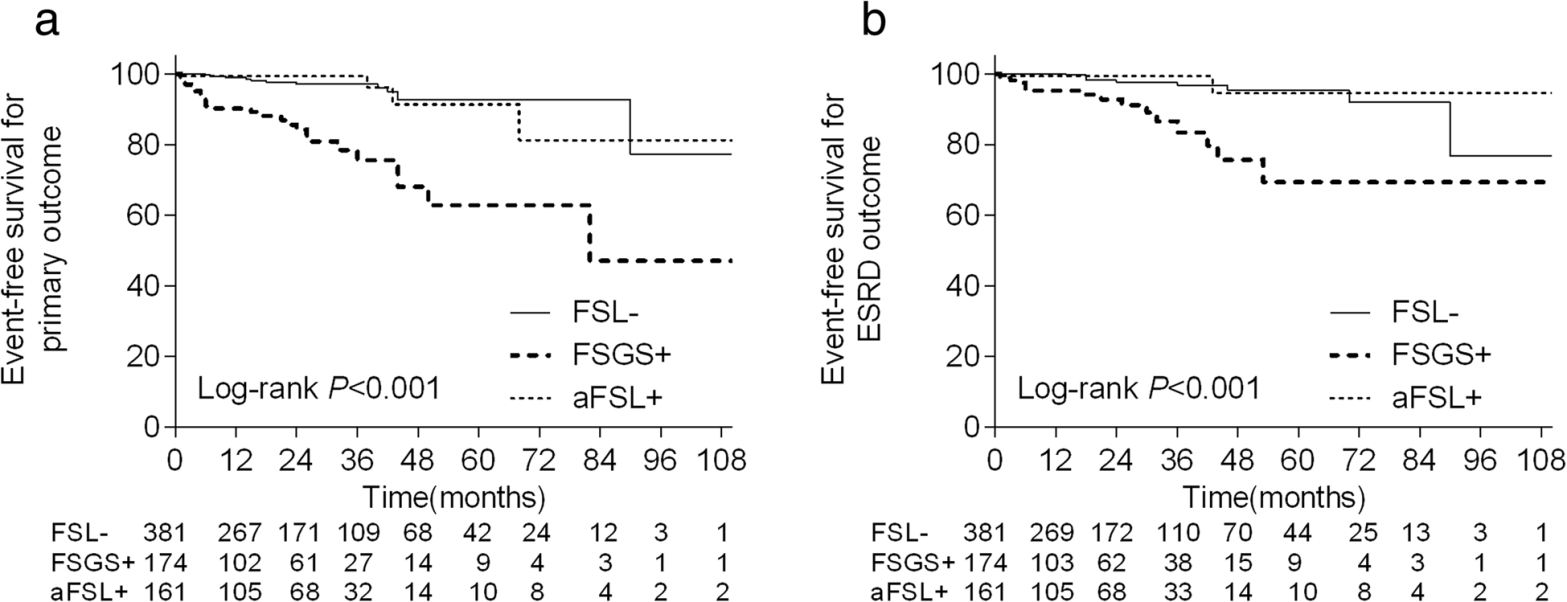
Kaplan-Meier survival curves were constructed for comparison of renal survival rates,campared with the FSL^-^ and aFSL^+^ groups, the IMN patients with FSGS had a significant higher risk progressing to primary (**a**) (both *P* < 0.001) and ESRD (**b**) (vs. FSL^-^ group: P < 0.001,vs.aFSL^+^ group: *P* = 0.001) outcome

**Fig. 3:**
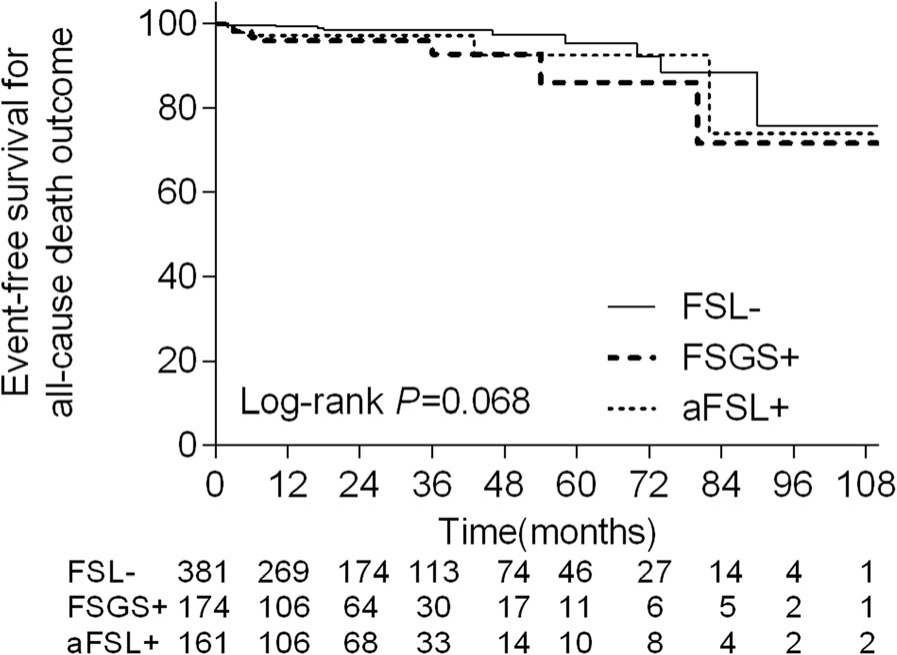
Comparison of all-cause death outcome between the three groups. The IMN patients with FSGS had a significant higher risk progressing to all-cause death outcome(*P* = 0.018), compared with the FSL^-^ groups, while the FSGS^+^ group and aFSL^+^ group had no significant difference(*P* = 0.544)

The IMN patients with FSGS had a significantly higher risk of progressing to the primary outcome and ESRD outcome compared with patients in the other groups (Fig. 2). The IMN patients with FSGS also had a significantly higher risk of progressing to the all-cause death outcome compared with the FSL^−^ groups, while the FSGS^+^ group and aFSL^+^ group had no significant difference (Fig. 3). At the 24-months follow-up point, the event-free survival for primary outcome in the FSGS^+^ groups was 84.2 ± 3.5%, compared with 99.4 ± 0.6% in the aFSL^+^ group and 97.2 ± 1.1% in the FSL^−^ group.

**Fig. 4:**
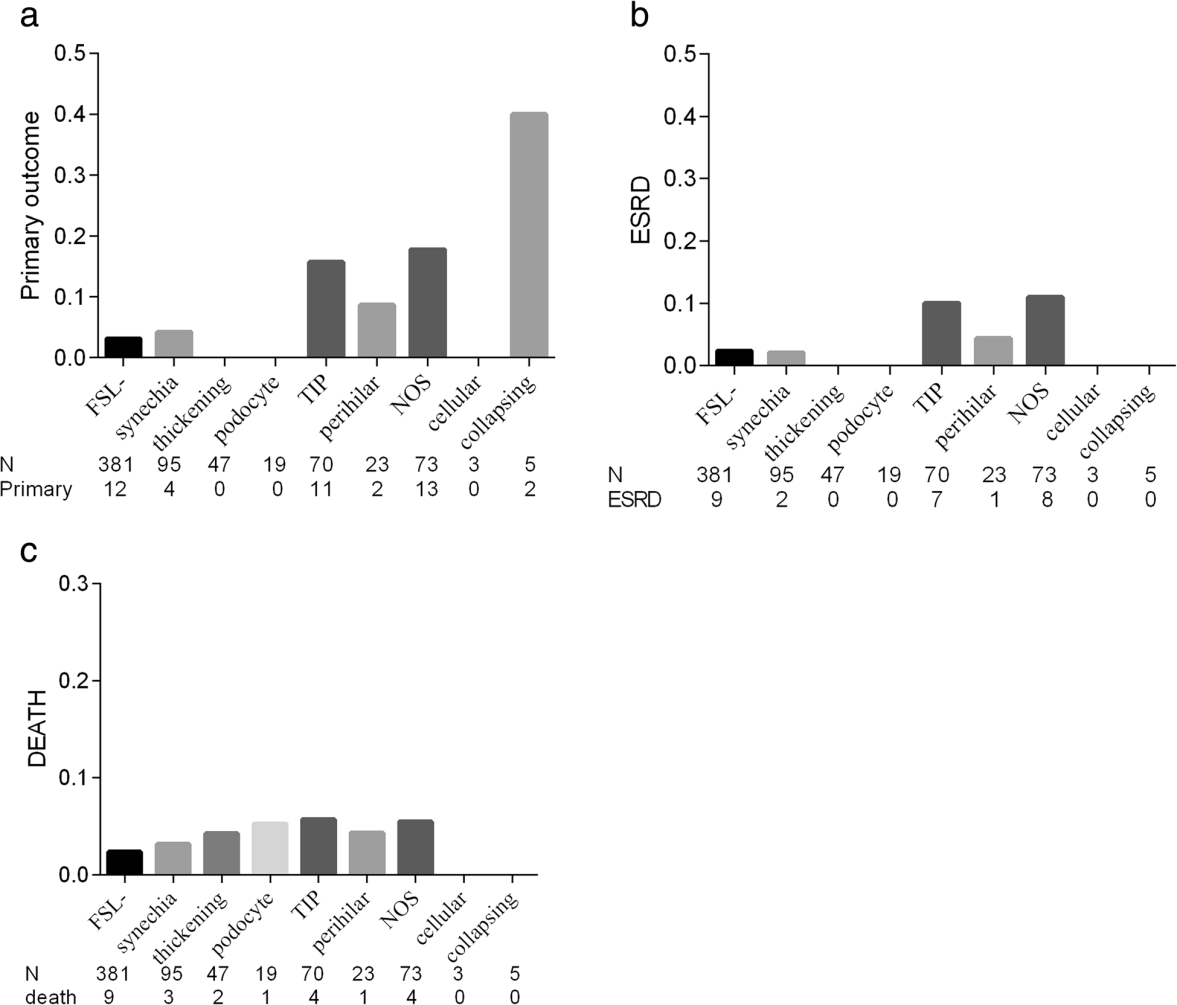
Patient outcomes in different classification of segmental lesion. **a** Ratio of the patients progressed to primary outcome. **b** Ratio of the patients to ESRD. **c** Ratio of the patients to all-cause death. Abbreviations: TIP, tip variant; NOS,FSGS not otherwise specified; podocyte, hyperplasia of podocyte

The patients with NOS, tip variant and collapsing variant had a higher ratio of progression to the primary outcome (Fig. 4a), and those with NOS, and tip variant had a higher ratio of progression to ESRD (Fig. 4b) and a numerically higher ratio of progression to all-cause death (Fig. 4c).

## Discussion

Focal segmental lesions, including typical FSGS lesions and atypical focal segmental lesions, are common in IMN. The percentage of IMN patients with FSGS lesions ranges widely different, and the predictive value of FSGS for worse renal outcome in IMN patients remains debated. In this retrospective IMN cohort from a single Chinese centre, we attempted to differentiate atypical FSL, named aFSL, from typical FSGS. Patients with aFSL, along with patients without FSL, had a better outcome with respect to progression to the primary outcome. In the current study, focal segmental glomerulosclerosis, excluding the atypical lesion, is a predictor of ESRD or eGFR decline of ≥50% of baseline eGFR in patients with idiopathic membranous nephropathy.

In 2004, D’Agati proposed a working classification of FSGS, including five types of lesions [9]. However, some segmental lesions, named aFSL by the present authors, comprising synechia, capillary shrinking, and GBM thickening involving the segmental glomerular tuft, which are unaccompanied by any typical FSGS lesion, were not included in the 2004 classification. Ehrenreich and Churg first reported focal sclerosis lesions in 30% of cases of MN [3]. The percentage of FSGS^+^ in IMN differed significantly in later studies (Table 4), different definitions in clinical practice may be one of the most important reasons. The present study attempts to separate aFSL from FSGS in IMN.

**Table 4:** Percentage of IMN patients with FSGS+ in the literature

Author	Percentage of FSGS (%)	Year of publication
Van Damme B et al. [4]	33(FSSH), 53(adhesion)	1990
Dumoulin A et al. [7]	41.7	2003
Shiiki H et al. [10]^a^	5.0	2004
Heeringa SF et al. [12]	41.5	2007
Gupta R et al. [19]	12.8	2010
Sprangers B et al. [13]	20.5	2012
Chen Y et al. [15]	10.1	2014
Morita M et al. [20]	10.4	2015
Gu QH et al. [14]	2.5	2016
Present study	22.5(aFSL), 24.3(FSGS)	/

Abbreviations: *FSSH* Focal and segmental sclerosis and hyalinosis, *aFSL* Atypical focal segmental lesion; ^a^Present in ≥20% of total glomeruli;

In an animal model, Wharram found that 21 to 40% depletion of podocytes showed capsular adhesions, mesangial expansion, and FSGS, and a > 40% depletion showed segmental-to-global glomerulosclerosis [21]. Subepithelial immune deposits may contribute to disrupting the podocyte attachment to the GBM [7],and the detachment of the epithelium may lead to focal sclerosis [4]. Gupta supported the hypothesis of glomerular hyperperfusion and hyperfiltration as one of the causative mechanisms in the development of FSGS in IMN [19]. Moritare ported that glomerular capillary injury was more prominent in MN combined with FSGS and that possible mechanisms of glomerular capillary injury included glomerular hypertrophy, decreased VEGF expression of podocytes, and thickening of glomerular capillary walls [20]. Previous studies have shown that morphological FSGS lesions in preeclampsia and malignant hypertension are likely mediated by the combination of glomerular endothelial cell injury and podocyte injury [22, 23]. Daehn indicated that endothelial mitochondrial oxidative stress determines podocyte depletion in segmental glomerulosclerosis, and segmental glomerulosclerosis, which develops as a result of podocyte-endothelial crosstalk [24].

The segmental podocyte disease and proliferation of the matrix may be considered a progression to FSGS in IMN, and this progression is reversible if appropriate measures are taken. If detachment or damage of the podocyte persists and deteriorates, aFSL may progress irreversibly to FSGS and renal injury. However, the synechia lesions may arise through physical stress placed on the tuft in the setting of severe nephrotic syndrome by the flux of protein-rich filtrate towards the tubular pole. This process is similar to that in the tip lesion of primary FSGS [25]. The phenomenon comprising high proteinuria and low levels of serum albumin was observed in IMN patients with aFSL, which supports this assumption. Smeets also reported that lesions detected by parietal epithelial cell markers were small and often located close to the glomerular tip in primary FSGS [26].

Unlike most previous studies that excluded patients followed up for less than 12 months [7, 11] or 6 months [13], the present study included patients followed up from 1 to 12 months,. This enrolment method was based on the finding that 10 of 24 patients died and 16 of 44 patients progressed to the primary outcome within the first 6 months in our study. Consistent with previous research, in the present analysis, older age [10, 15], hypertension [13], eGFR at biopsy [10, 12, 13, 15, 27, 28], FSGS [4, 7, 8, 15], chronic tubulointerstitial injury [8, 10, 15, 27–30], global Glomerulosclerosis [10], and vascular lesion [7, 8] were predictors of IMN progression to ESRD or eGFR decline of > 50% of baseline eGFR, or doubling of creatinine. In some previous studies, the FSGS lesion in IMN seemed to have a limited predictive value; when combined with clinical and other pathological parameters, it did not emerge as an independent prognostic factor for the decline in renal function [11, 12], which was consistent with our observations in the model 1 mutivariate analysis. However, we noticed that the reported percentage of IMN patients with FSGS lesions differs significantly between studies, and we found that after excluding the atypical lesion, FSGS lesion was an independent predictive factor in IMN for progression to a 50% decline in the initial estimated glomerular filtration rate or ESRD in the current study.

There were some limitations to this study. First, an accurate definition of aFSL is still debated, and the adhesion and hyperplasia of podocytes may emerge as different mechanisms. However, the classification used in this study stressed the characteristics differentiating atypical FSL from typical FSGS in IMN. Second, this study used a retrospective design, and the effect of confounding factors could not be fully excluded. Some important baseline covariates may also not have been equally distributed. Third, this analysis relied on data from a single centre. Moreover, a longer follow-up duration could have better validated our findings, and the patients who were lost to follow-up could have affected this study’s outcomes. Data over time, such as change in proteinuria as a prognostic factor, could not easily be obtained in this retrospectively studied population.

## Conclusions

Histopathologic findings tubulointerstitial fibrosis area ≥ 15% and FSGS at biopsy were independent risk factors for the primary outcome, even when combined with clinical parameters. Focal segmental lesions in IMN were common in the present study, which is the first, to our knowledge, to differentiate aFSL from FSGS in IMN and to show that FSGS, excluding the aFSL, was an independent predictor for a 50% decline in initial eGFR or ESRD.

## Data Availability

The datasets used during the current study are available from the corresponding author on reasonable request.

## Abbreviations

ACE: Angiotensin-Iconverting enzyme
aFSL: Atypical focal segmental lesions
CI: Confidence interval
CKD: Chronic Kidney Disease
eGFR: Estimated glomerular filtration rate
ESRD: End stage renal disease
FSGS: Focal segmental glomerulosclerosis
FSL-: Without focal segmental lesions
HR: Hazard ratio
IMN: Idiopathic membranous nephropathy
KDIGO: Kidney disease improving global outcomes
